# PPSW–SHAP: Towards Interpretable Cell Classification Using Tree-Based SHAP Image Decomposition and Restoration for High-Throughput Bright-Field Imaging

**DOI:** 10.3390/cells12101384

**Published:** 2023-05-13

**Authors:** Polat Goktas, Ricardo Simon Carbajo

**Affiliations:** 1 UCD School of Computer Science, University College Dublin, Belfield, D04 V1W8 Dublin, Ireland; 2 CeADAR: Ireland’s Centre for Applied Artificial Intelligence, Clonskeagh, D04 V2N9 Dublin, Ireland

**Keywords:** artifact, bright–field images, cell therapy, image decomposition, image restoration, interpretable machine learning, Shapley additive exPlanations, stem cells

## Abstract

Advancements in high–throughput microscopy imaging have transformed cell analytics, enabling functionally relevant, rapid, and in–depth bioanalytics with Artificial Intelligence (AI) as a powerful driving force in cell therapy (CT) manufacturing. High–content microscopy screening often suffers from systematic noise, such as uneven illumination or vignetting artifacts, which can result in false–negative findings in AI models. Traditionally, AI models have been expected to learn to deal with these artifacts, but success in an inductive framework depends on sufficient training examples. To address this challenge, we propose a two–fold approach: (1) reducing noise through an image decomposition and restoration technique called the Periodic Plus Smooth Wavelet transform (PPSW) and (2) developing an interpretable machine learning (ML) platform using tree–based Shapley Additive exPlanations (SHAP) to enhance end–user understanding. By correcting artifacts during pre–processing, we lower the inductive learning load on the AI and improve end–user acceptance through a more interpretable heuristic approach to problem solving. Using a dataset of human Mesenchymal Stem Cells (MSCs) cultured under diverse density and media environment conditions, we demonstrate supervised clustering with mean SHAP values, derived from the ‘DFT Modulus’ applied to the decomposition of bright–field images, in the trained tree–based ML model. Our innovative ML framework offers end-to-end interpretability, leading to improved precision in cell characterization during CT manufacturing.

## 1. Introduction

Cell Therapies (CTs), a cutting–edge treatment modality, have significant clinical potential for treating a variety of disease targets including cancer, degenerative conditions such as osteoarthritis, and immunological disorders such as Crohn’s disease [1,2]. Mesenchymal Stem Cells (also referred to as mesenchymal stromal cells, MSCs) are one of the most widely investigated clinical CTs, possessing the ability to reduce inflammation and stimulate tissue repair in various inflammatory disorders, primarily due to their paracrine activity [3,4,5]. However, these therapeutics present unique manufacturing challenges that include donor variability, tissue source, and media environment differences [6,7,8]. To improve manufacturing, recent advancements in imaging and Artificial Intelligence (AI) technology aim to provide quick, relevant insights into CT manufacturing to enhance bioprocess analytics [9,10,11].

Numerous researchers have explored various strategies to address the challenges of CT manufacturing. For instance, Imboden et al. [9] examined the heterogeneities of live MSCs using AI–driven label–free imaging, while Zhang et al. [10] utilized deep learning for label–free nuclei detection from implicit phase information of MSCs. Kim et al. [11] conducted high–throughput screening of MSC lines using deep learning techniques. These investigations highlight the increasing interest in harnessing AI and imaging technologies to enhance CT manufacturing. While there have been research efforts to explore deep learning and machine learning (ML) models through various visualization techniques and feature extraction at different network points [12,13], these approaches often focus on gaining insights into the biology behind the analytics and developing techniques suitable for smaller sample sizes. For example, Yosinski et al. [14] investigated the transferability of features in deep neural networks, and Olah et al. [15] presented building blocks for interpretability in deep learning models. However, the interpretation of AI models applied in cell analytics has predominantly been confined to assessing the entire model via global feature analysis, neglecting the relative significance of individual prediction features. This approach falls short in thoroughly understanding each prediction and the crucial factors influencing it. Our proposed workflow aims to address these limitations by providing a more comprehensive understanding of the underlying biology and offering a methodology that is applicable even to smaller sample sizes.

Additionally, while the progress in high–throughput microscopy screening has contributed to the development of promising automated image analysis tools by enhancing the accessibility and availability of imaging technologies, background noise in biological samples remains a significant obstacle to retrieve faint details from the raw data that are close to the intrinsic noise [16]. Moreover, factors such as camera exposure, axial misalignment and tilt effects on the lens [17], dust, non–uniform light sources [18,19], and vignetting (or shading) artifacts [20] cause uneven illumination of the scene or specimen in the microscope. In bright–field (BF) imaging, specific type of artifacts, i.e., vignetting artifacts, are frequently seen. These are characterized by a decrease in brightness or contrast from the center of the image towards the outer regions [21]. Kevin et. al. noted that uneven illumination, often misperceived, leads to biased results in many studies [22]. Accordingly, illumination correction is a key component for both the setup of image acquisition and the processing of high–content images for analysis [23,24,25]. These artifacts degrade image quality and can lead to false–negative findings in AI models. Traditionally, AI models have been expected to learn to deal with these artifacts; however, success in an inductive framework relies heavily on the availability of sufficient training examples. In many cases, the potential impact of artifact structures, particularly vignetting and non–uniform illumination, has been ignored, specifically for the performance assessment of a trained AI model. However, in practice, faster cell assays and label–free interpretable quantitative approaches to measure the therapeutic quality attributes of CTs are required, which can be utilized to further refine and enhance cell manufacturing processes toward experimental endpoints.

In this study, we aimed to overcome the limitations mentioned earlier by developing a label–free analysis workflow that leverages the information from BF images for the functionally relevant manufacturing characterization of human MSC expansion. We propose a workflow that addresses two major challenges in the field: (1) reducing noise in the data, which can lead to more accurate predictions, and (2) developing easy-to-interpret features to aid end–user interpretation. Our workflow integrates advanced noise reduction techniques with feature engineering, resulting in a supervised learning algorithm for label–free cell density and media classification in human MSC cultures. This approach not only improves the quality of the data but also provides interpretable insights for end–users.

**Figure 1:**
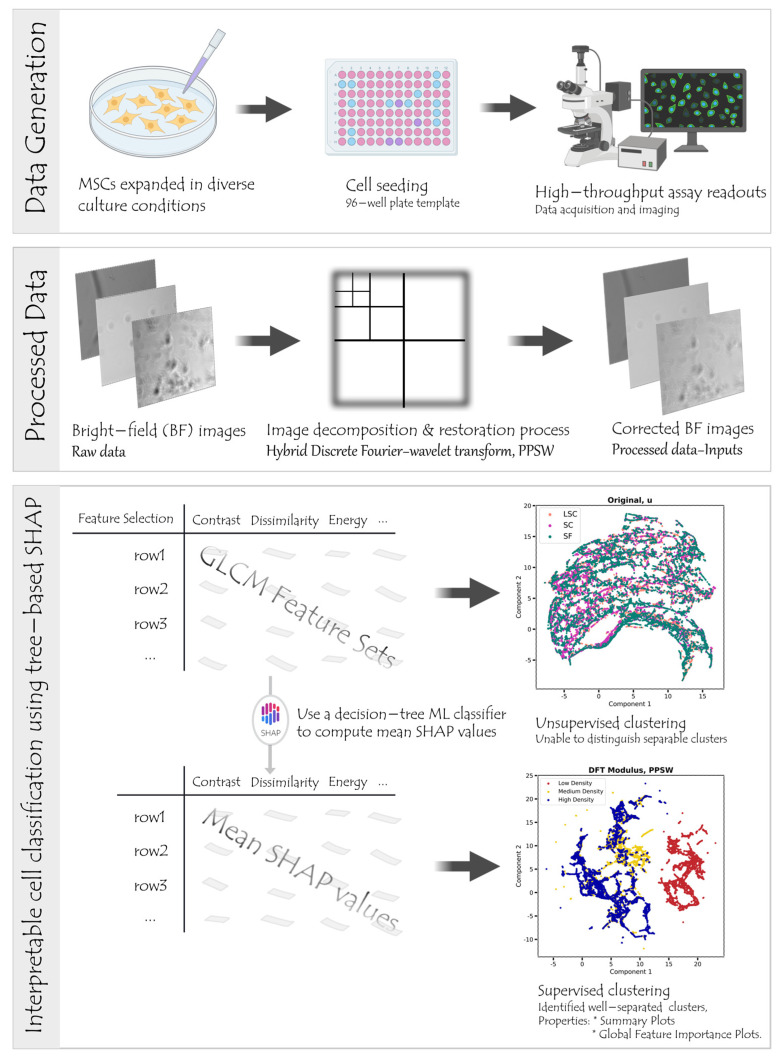
Our label–free cell density and media classification workflow. First, the bright–field images of Mesenchymal Stem Cells (MSCs) were captured using the Perkin Elmer Operatta system equipped with 20× magnification lenses. The images were then pre–processed, using an image decomposition and/or restoration technique to reduce the possible adverse impact of sample variability on classification accuracy, including artificial structures in label–free images. We extracted Gray–level Co–occurrence Matrix (GLCM) texture feature parameters from pre–processed data and utilized supervised machine learning models for classification. Once the classifier was trained, it was used to classify the state of unlabeled cells, such as cell density or media environment conditions. To assess the significance of texture features in machine learning models, we used model–agnostic permutation importance and feature importance scores for the optimal pipeline scenario. The relative feature importance was also provided by the Shapley Additive exPlanations (SHAP) TreeExplainer multi–classification utility. Finally, average SHAP values were used to generate supervised clustering to identify better–separated clusters utilizing a more structured representation of the Gradient Density dataset. The graphic component of ‘Data Generation’ was created with BioRender.com (accessed on 26 February 2023). The cluster plot titled ‘Original, u’ represents an unsupervised clustering attempt, which was not successful in distinguishing between three classes. The supervised plot, on the other hand, demonstrated improved classification using our proposed workflow. Further details regarding the ‘Original, u’ plot and the supervised plot can be found in Section 2.

**Figure 2:**
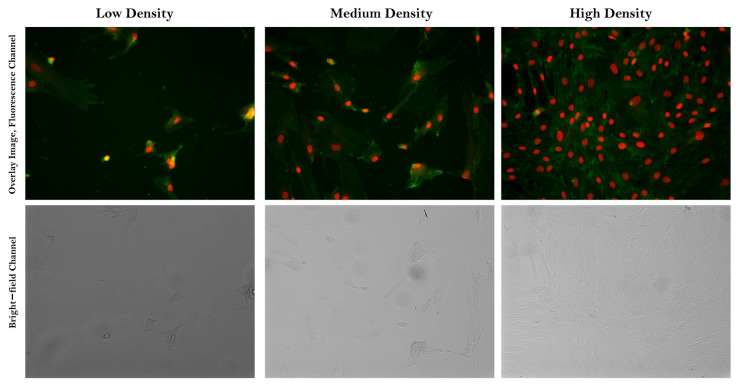
Representative overlay and bright–field images of Low (**left**), Medium (**center**), and High (**right**) density spectra in the Gradient Density samples, captured by a Perkin Elmer Operetta microscope using 20× magnification lenses. Overlay images display high–dimensional cellular features (green, Cell mask) and nucleus features (red, Hoechst).

This workflow is applied to BF and fluorescent channel images of human MSCs, which were cultured in various media under different bioprocess conditions (Figure 1 and Supplementary Materials Figure S1 for workflow diagrams and Figure 2 and Supplementary Materials Figure S2 for representative overlay and BF images). During image acquisition, we recorded the experimentally determined target density and media conditions as labels. To minimize the potential impact of artifact structures on classification performance, we pre–processed BF images using image decomposition and/or restoration techniques. We then extracted six Haralick texture feature sets from the pre–processed data, including Gray–level Co–occurrence Matrix (GLCM) attributes: Angular Second Moment (ASM), Contrast (CON), Correlation (COR), Dissimilarity (DIS), Energy (ENR), and Homogeneity (HOM) [26]. These features were chosen due to their proven effectiveness and generalizability in previous research [27]. They have been found to be generalizable across various biological datasets [28], and we anticipate that they would translate well to other datasets with similar characteristics. These image features served as input for supervised ML models, enabling us to classify cell density and media environment conditions without using any stains.

For the optimal pipeline scenario, we trained decision–tree classifiers and evaluated model–agnostic permutation importance and global feature importance scores to assess texture feature relevance. We also determined the average Shapley Additive exPlanations (SHAP) values using the SHAP TreeExplainer [13] to identify which features contribute the most to specific class assignments, thus enhancing ML interpretability. Finally, we conducted dimensionality reduction based on SHAP values to better separate clusters within the label–free imaging dataset. Our approach demonstrates the potential of label–free imaging with interpretable ML models to improve MSC classification and characterization in CT manufacturing. The combination of image pre–processing techniques and feature extraction from high–throughput BF images allows for the efficient identification of key factors that influence MSC expansion without relying on staining procedures. By employing tree–based SHAP values, we were able to determine which features contribute most significantly to class assignments, improving the interpretability of our ML models. By addressing the limitations of existing approaches and providing a rapid and simple method for identifying MSCs in CT manufacturing, our work advances the state-of-the-art in this field.

Future work in this area may explore the integration of additional imaging modalities and machine learning algorithms to further enhance the accuracy and interpretability of MSC classification in CT manufacturing. Moreover, the scalability of our approach could be tested on more complex and larger datasets, with the goal of generalizing these techniques to other types of CTs. Finally, further optimization of pre–processing and feature extraction methods may lead to even more accurate and efficient identification of MSCs, ultimately contributing to the development of better CT manufacturing processes.

## 2. Materials and Methods

### 2.1. Data Collection

Traditional human MSCs used in basic research, and in most clinical trials, are cultured in basal media supplemented with fetal bovine serum (FBS) and growth factors [29]. The development of serum–free (SF) and chemically used media environments for the clinical production of human MSCs is a high priority in the CT manufacturing area [30,31]. To evaluate the effectiveness of image decomposition and/or restoration techniques for high–throughput BF images, we used a non–publicly available Gradient Density dataset of human MSCs. This dataset was generated and provided by Stem Cell R&D at the Regenerative Medicine Institute (REMEDI NUI, Galway, Ireland). The dataset consists of fluorescent readouts from Hoechst dye staining and high–throughput BF images derived from human MSC samples. It is important to note that our study did not directly involve human samples; rather, we utilized data in the form of cell images from previously characterized human cell lines. These cell lines were obtained and characterized by Stem Cell R&D during prior studies, which received full ethical approval from the NUIG Research Ethics Committee. As our study did not involve the direct sampling of human cells, we did not require additional ethical approval for this research. We encourage interested researchers to contact the Stem Cell R&D at REMEDI NUI Galway for potential collaboration or data access opportunities related to the Gradient Density dataset.

Bone marrow–derived human MSCs were expanded under three different media conditions: SF medium, serum–containing (SC) medium (a combination of media + 10% FBS + 1 ng/mL fibroblast growth factor 2), and low–serum containing (LSC) medium containing 2% FBS. These cells were then seeded at three different densities: low, medium, and high, ensuring an equal distribution of images for each targeted medium and density environment to avoid any classification imbalance. Images were captured in confocal mode at 20× magnification on an Opera Phenix high–content screening system (Perkin Elmer Operatta, Waltham, MA, USA). During image acquisition, we produced raw images of 1360 × 1024 pixels at 16 bits per channel per pixel and documented the target density and media conditions determined experimentally for the labels. For our experimental scenario, six fields per well were captured for each type of cell media condition, resulting in a total of 1152 images.

### 2.2. Image Decomposition and/or Restoration Techniques

**Figure 3:**
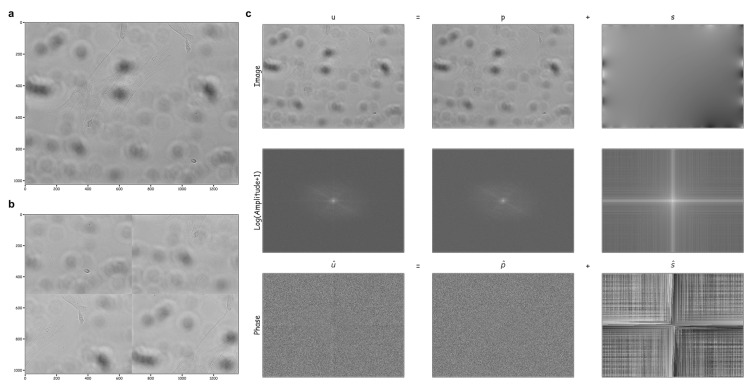
An illustration of the periodic plus smooth image decomposition (PPSD) technique applied to an unevenly illuminated bright–field image captured with 20× magnification lenses on a Perkin Elmer Operatta microscope. Assuming that the original image (**a**) was periodic, its quadrant–swapped version (**b**) would be identical to the original, as seen in the Discrete Fourier Transform (DFT), enabling the identification of cross–structure artifacts along the original image borders. The representative image was decomposed into its periodic and smooth components (**c**, top row). The DFT modulus (or magnitude) component (**c,** middle row, in the log scale) and the phase component (**c,** bottom row) illustrated the Fourier spectrum throughout all images.

We investigated the Periodic Plus Smooth Image Decomposition (PPSD); a Discrete Fourier Transform (DFT) approach that decomposes an image domain *u* into its periodic component *p* and its smooth component *s* to avoid the cross–structure artifact spectrum across the original image border (Figure 3). The ‘DFT Modulus’ property of the image was computed in log scale (Figure 3c; middle row). The cross–structure artifact spectrum disappeared in the Fourier transform of *p* while the others remained unchanged. We also examined the robust principal component analysis (robust PCA) in which the raw data were decomposed into a low–rank component and a sparse matrix component [32]. This reduced the dimensionality of the data based on the low–rank structure and the sparsity of the outliers; however, this method was computationally unfeasible for a high dimensional imaging dataset.

**Figure 4:**
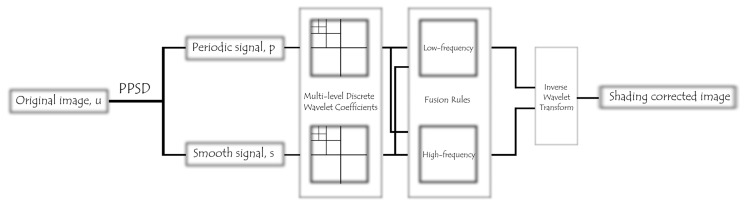
Conceptual illustration of the periodic plus smooth wavelet transform (PPSW) technique applied to bright–field images, encompassing the multilevel decomposition and reconstruction processes of the wavelet transform.

Here, we proposed the Periodic Plus Smooth Wavelet Transform (PPSW), an image decomposition and restoration technique that properly enhances the quality of structured background components on the BF images by minimizing the impact of vignetting (or shading). We first computed the periodic component *p* and the smooth component *s* of the original image through the PPSD technique and then decomposed each image component using a discrete Wavelet transform with the multi–level wavelet filter. Finally, we reconstructed the fused decomposed images to create an image with uneven illumination adjusted (Figure 4). For improved image reconstruction, selecting the appropriate wavelet function was crucial in our image decomposition and restoration technique. Determining the wavelet type and the number of decomposition levels was vital to ensure success. This study utilized discrete Wavelet transform types, including Biorthogonal, Coiflets, Daubechies, and Symlets mother wavelets, as depicted in Supplementary Materials Figure S3. Initially, we evaluated the classification accuracy of cell density and media conditions using ten second–level mother wavelet functions in Random forests, the top–performing classifier in our experimental trials. We realized that the bior1.1 second–level mother wavelet type performed better than all other wavelet competitors of concern in this study, regardless of density and media contexts (Supplementary Materials Tables S1 and S2). Following that, the classification performance of bior1.1 multi–level mother wavelet types (up to fifth–level decomposition) was evaluated using Random forest classifiers. The results showed that the classification accuracy for cell density varied from 92.91 to 94.75% and for media environment conditions, from 88.64 to 91.43%. Therefore, we utilized the bior1.1 mother wavelet type with a fifth–level decomposition in our proposed image decomposition and restoration technique throughout this work.

### 2.3. Feature Engineering

We performed a GLCM–based texture feature analysis to classify sources of variability from high–throughput BF images, such as cell culture density or media environment conditions, in the human MSCs. The choice of GLCM–based texture feature analysis was influenced by its well–established position in the field of image processing and computer vision, along with its proven effectiveness and robustness in capturing texture information and distinguishing different types of patterns in images [26,33,34]. The GLCM is a square matrix whose entries represent the probability of gray–level co–occurrences at a specific distance and orientation in the region of interest. To compute Haralick features from the GLCM that counts the co–occurrence of neighboring gray levels in the BF image, we first dispersed the pixels in an image over a specific distance d and oriented them in a particular orientation θ, representing a distinct relationship between the neighboring and reference pixels.

We selected three main orientations (0, 45, and 90 degrees) and five distances, ranging from 1 to 5 pixels, to compute the following six features: Angular Second Moment (ASM), Contrast (CON), Correlation (COR), Dissimilarity (DIS), Energy (ENR), and Homogeneity (HOM). These six features were chosen based on their significant discriminative power and their ability to effectively represent various aspects of texture information present in the images, such as homogeneity, linear dependence, spatial variation in pixel intensity values, and regional similarity in our specific dataset. Haralick et al. described the above-mentioned parameters of the GLCM [26]. Mainly, ASM and COR measure the texture homogeneity and linear dependence of neighboring gray levels in the image, while CON measures spatial variation in pixel intensity values [26,33,34]. ENR is derived from ASM. High ASM and COR values indicate the likelihood of a linear relationship between the gray levels of adjacent pixels. Conversely, gray–tone large local variation dependence of the image has higher values of CON. Additionally, DIS is analogous to CON and is inversely related to HOM, which is a measure of regional similarity in the image [26,33,34].

### 2.4. Building and Evaluating Machine Learning Classification Models

The data were partitioned into training and test datasets with the ratio of 7:3 by random sampling using Phyton 3.91.12 sklearn.model_selection (version 1.1.1 of scikit-learn). We applied a variety of ML methods during the multi–class classification, including a decision tree [35,36], random forest [37], adaptive boosting (AdaBoost) [38], gradient–boosting via XGBoost (version 1.6.1) [39], a gradient–boosting decision tree (GBDT) [40], a histogram–based gradient–boosting classification tree (HistGBDT) [41], and a light gradient–boosting machine (version 3.3.3 of LightGBM) [42] with specified hyperparameters. Specifically, decision trees are flowchart–like structures used for both classification and regression tasks, where each internal node represents a feature, each branch represents a decision rule, and each leaf node represents an outcome [35,36]. Random forest is an ensemble learning method that constructs a multitude of decision trees during training and outputs the mode of the classes of the individual trees for classification tasks [37]. XGBoost is an optimized distributed gradient–boosting library designed to be highly efficient, flexible, and portable [39]. LightGBM is a gradient–boosting framework that uses tree–based learning algorithms, which is designed to be distributed and efficient with a focus on training speed and higher efficiency [42].

These methods aim to assess the ability to classify stem cell–manufacturing characteristics from high–throughput BF images using image decomposition and/or restoration techniques, along with six Haralick texture feature sets, in order to determine the optimal pipeline configuration. To guard against overfitting, we retained a test dataset for model evaluation and ensured that it was not used for algorithm tuning. In our preliminary experiments, we attempted feature standardization using ‘sklearn.preprocessing.StandardScaler’ during the pre–processing stage and explored the use of k–fold cross–validation. However, we found that neither feature standardization nor k–fold cross–validation provided significant improvements in the model’s performance. As a result, we decided not to include these techniques in our final analysis to save computational effort while maintaining reliable results. Our methodology, which involved retaining a test dataset and not using it for algorithm tuning, effectively guards against overfitting and ensures the robustness of our ML models. Four classification metrics, the accuracy and macro average of precision, recall, and the F1–score, were used to evaluate the performance of the ML classifiers in our experimental scenario. The model with highest values was then used to determine the optimal pipeline components of interest for the model–building process overall after evaluation with the testing dataset. All experiments were conducted on a Graphic Processing Unit (GPU) Sever (2x NVIDIA Ampere A100 PCle, 250W, 40GB Passive: 2x Intel Xeon Gold 6252 2.1G, 24C/48T, 10.4GT/s, 35.75M Cache, Turbo, HT (150W) DDR4-2933; 12x 32GB RDIMM, 3200MT/s, Dual Rank) and a Laptop computer (Dell Precision 5550: Intel^®^ Core™ i7-10850H CPU @2.70 GHz 2.71 GHz RAM 32.00GB Windows 10 Pro).

### 2.5. Feature Importance Measures

We employed model–agnostic permutation importance and feature importance scores [37,43] for the case of the optimal pipeline scenario to assess the relevance of texture feature sets in the trained ML model. This approach is widely employed in the field due to its flexibility, robustness, and capability to provide a consistent evaluation of feature importance across different types of models irrespective of their internal structure or algorithmic design. The relative feature importance was generated using the Shapley Additive exPlanations (version 0.41.0 of SHAP) TreeExplainer multi–classification utility, which computes Shapley values to quantify the contribution of each feature to the prediction for individual instances. These SHAP values, derived from the test data, can be thought of as a latent representation of the Gradient Density dataset, emphasizing the most relevant parameters for the characterization of stem cell manufacturing in the trained ML model. This approach allows us to maintain a balance between the interpretability of the ML model and its efficiency.

### 2.6. Dimensionality Reduction Based on SHAP Analysis

Uniform Manifold Approximation and Projection (UMAP) [44] was utilized to compress the raw data or SHAP values with the trained model to a two–dimensional space in order to improve the classification performance of the stem cell manufacturing characterization from label–free images. Unlike alternatives, such as t–SNE [45], UMAP maintains both the local and global structure of the data, which is a significant advantage for reducing data dimensionality. SHAP values, which provide a measure of feature importance for each data point, can be used to guide clustering in a supervised manner. By applying clustering techniques directly to SHAP values, we can reduce the noise from irrelevant features and weight the data by a measure of relevance that emphasizes the most important aspects. In contrast, traditional clustering biases inputs based on the amplitude of their distributions, making it challenging to cluster meaningful structures for dimensionality reduction.

To demonstrate the benefits of dimensionality reduction based on SHAP values, we conducted supervised clustering by utilizing average SHAP values from high–throughput BF images, combined with various Haralick features from image decomposition and/or restoration techniques, through the use of Random forest classifiers. This advanced application of SHAP values in our analysis allows for a more effective and interpretable dimensionality reduction, ultimately enhancing the performance of our classification models.

### 2.7. Statistics

The Kolmogorov–Smirnov test was used to evaluate the normality of the continuous variables [46]. Unless otherwise specified, descriptive statistics were provided as the mean (standard deviation) and median (interquartile range; 25–75%) for descriptions of normally and non–normally distributed data, respectively. For our dataset, we focused on the median (interquartile range; 25–75%), peak signal-to-noise ratio (PSNR), and dB values obtained from image decomposition and/or restoration techniques. We performed a non–parametric technique with the Kruskal–Wallis test [47] to compare the values of each GLCM texture feature set across Low, Medium, and High density samples in the experimentally established target media environment. In order to compare the image decomposition and/or restoration techniques for each class cell density assignment, we computed the mean of differences (=bias) between the ‘DFT Modulus’ transform applied to the decomposition of images and the original BF images using average SHAP values from random forest classifiers. The significance level was defined as *p* < 0.05, as indicated by Asymptotic Sig. (2–tailed). All statistical analyses were performed using IBM SPSS Statistics 27 (IBM Corp., Armonk, NY, USA).

## 3. Results

### 3.1. Computing a Hybrid Discrete Fourier–Wavelet Transform Approach and Assessing Anomalies

We examined a discrete Fourier transform approach, named the Periodic Plus Smooth Image Decomposition (PPSD), to decompose an image domain into the sum of a ‘periodic’ component, which expresses the majority of the image information but has no edge effects, and a ‘smooth (or harmonic)’ component, which consists of smooth fluctuations at the borders of the image [48]. Here, we validated its usage to discriminate strong discontinuities across the image border for label–free stem cell imaging datasets. In practice, since the contents of microscopy images are not periodic, the discrete Fourier transform approach allows us to estimate a cross–structure artifact spectrum in our image domain. As shown in Figure 3, the periodic component of the representative original image (*p*) closely resembled the spectrum of the original image (*u*) but avoided edge effects across the image border, whereas the smooth component of the image (*s*) often collected border discontinuities caused by the periodicity of *u*. Accordingly, it is possible that the artifact faded away in the Fourier transform of *p* (particularly as illustrated in the phase spectrum diagram), but the other portion of the spectrum remained constant, with slight changes in fluctuations in the image domain.

**Figure 5:**
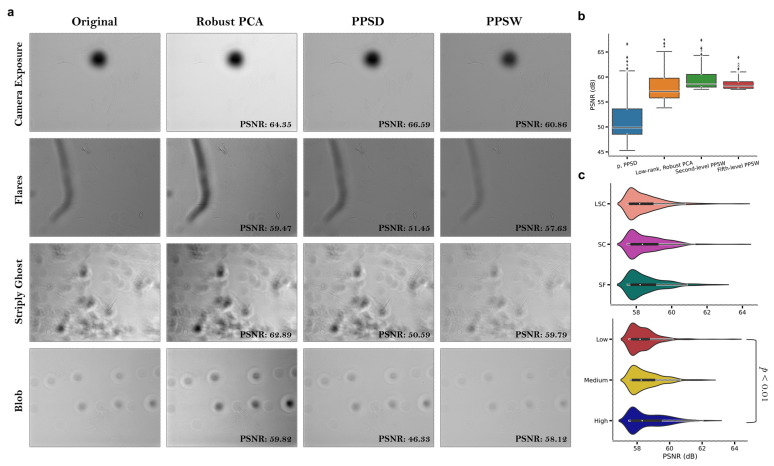
Pre–processed bright–field (BF) image examples with decomposition and/or restoration techniques. (**a**) Common artifacts in label–free images, with each row showing a specific category: original image (1st column), robust PCA–based low–rank matrix (2nd column), periodic component via PPSD (3rd column), and corrected BF image using PPDW (4th column). Image quality metrics, including peak signal-to-noise ratio (PSNR, dB), were compared. (**b**) Boxplots of PSNR values for different techniques, showing median, interquartile range (25% to 75%), and outliers indicated as individual dots. (**c**) Violin plots for cell culture density (lower) and media environment (upper) using PSNR values from the fifth–level PPSW technique. Boxes display the mean (bold black line), median (white dot), and 75th and 25th percentiles (upper and lower lines), with surrounding kernel density plots. PSNR values represented by violin plots: LSC (soft red), SC (moderate magenta), SF (dark cyan); Low (strong red), Medium (vivid yellow), High (dark blue).

We next proposed a novel image decomposition and restoration technique called the Periodic Plus Smooth Wavelet Transform (PPSW) to reduce the effect of vignetting (or shading), which accurately improves the quality of structured background components in the label–free images. To evaluate the performance of our proposed image decomposition and restoration technique, we used a high–throughput stem cell imaging dataset for human MSCs and observed main artifact types including camera exposure (background variation as the numbers of objects in the image domain varies), lens flare, dust, blob–like, and ‘stripy ghosts’ artifacts on the BF images (Figure 5a). The ‘stripy ghost’ artifact is caused by sample movement or vibration during image acquisition, resulting in elongated, striped shapes that appear to follow the movement in the image domain. We first conducted a comprehensive quantitative evaluation of image decomposition and/or restoration techniques on the BF images in different media environments. Our proposed technique significantly mitigated the above–mentioned undesired light sources, making it easier to clarify the visibility of image features in the shaded regions while preserving the appearance of uneven illumination effects on the images using other image decomposition techniques (Figure 5a). We thoroughly analyzed the whole image dataset in terms of the PSNR, a metric commonly used to evaluate the quality of image reconstruction or noise–reduction techniques. PSNR measures the ratio between the maximum possible power of a signal and the power of corrupting noise that affects the fidelity of its representation. Higher PSNR values indicate better noise reduction performance. For our dataset, we focused on median (interquartile range; 25–75%) dB values obtained from image decomposition and/or restoration techniques, as described in Section 2, in comparison to original BF images. We found that the values were 57.108 (55.810–59.763) dB for low–rank matrix decomposition via Robust Principal Component Analysis (robust PCA) [32], 49.875 (48.526–53.679) dB for the periodic component of the original image via PPSD, and 58.589 (57.975–60.532) dB and 58.229 (57.716–59.056) dB for the bior2.4 mother wavelet type using second– and fifth–level decomposition via PPSW techniques, respectively (Figure 5b). Taking all results from the fifth–level PPSW technique into consideration, our image quality evaluation revealed a significant difference in cell culture density only between the Low and High gradient groups (*p* < 0.01, Figure 5c), but failed to consider all media conditions. Therefore, we believe that it is crucial to be able to employ texture feature patterns obtained from an image decomposition and/or restoration technique for label–free, high–throughput images in order to establish an ML model with highly differentiated clusters of MSCs corresponding to different cell culture and media conditions.

### 3.2. Achievement of a High Classification Accuracy with ‘DFT Modulus’

In our analysis, the descriptive statistics for the GLCM Homogeneity, Contrast, Correlation, and Dissimilarity features in BF images were found to be higher than those of the GLCM Energy and ASM features in all examined samples regardless of the media environment. The Kruskal–Wallis test indicated statistically significant evidence (p < 0.001) from Low to High density spectra for each target media environment in our original BF images (Table 1). During the establishment of our ML classification models, our investigation into the ‘DFT Modulus’ transform applied to BF images revealed substantially higher descriptive statistics. Therefore, we considered that it was crucial to employ the ‘DFT Modulus’ transform strategy for an ML model with high classification performance criteria for the label–free imaging dataset. Indeed, we were able to establish highly accurate tree–based ML models to reduce the possible adverse impact of sample variability on the classification accuracy of the cell culture and media conditions, including that of artifact structures on BF images (Supplementary Materials Tables S3 and S4).

Regarding the performance assessment of the multi–class classification in our experiment trials, random forests in our baseline scenario, using original BF images, achieved an average accuracy (AC) value of 80.129 and 80.146% for cell culture density and media environment conditions, respectively; false–positive cases would be tolerable but false–negatives are not acceptable for highly differentiated clusters of MSCs. Therefore, we examined the ‘DFT Modulus’ transform applied to the decomposition of BF images and found that there was a significant increase in the random forests with an AC value of 94.51 and 94.75% for the scenarios of the ‘DFT Modulus’ of the periodic component, PPSD, and the PPSW (the bior1.1 mother wavelet using fifth–level decomposition) techniques, respectively. In our experiments, the performance of gradient–boosting via XGBoost, light gradient–boosting machine (LightGBM), and decision tree models was comparable to that of random forests, with an AC value ranging from 88.15 to 91.49% for cell density and from 81.41 to 90.60% for the media environments. Furthermore, we discovered that the periodic component decomposition (PPSD) and our proposed decomposition and restoration technique (PPSW) yielded superior performance compared to the low–rank matrix and sparse decomposition achieved through robust PCA. Notably, robust PCA’s sparse decomposition demonstrated comparable performance to PPSD in the classification of the cell media environments. Detailed information on all tree–based ML models used in this study can be found in Table 2 and Table 3, as well as Supplementary Materials Tables S3 and S4. The decision–tree ML models enabled us to achieve superior performance for the classification of stem cell manufacturing characterization using high–throughput BF images with the periodic component, PPSD, and our proposed image decomposition and restoration, PPSW technique, utilizing six Haralick texture feature sets.

### 3.3. Supervised Clustering Based on SHAP Values Leads to More Accurate Cluster Analysis

We next attempted to identify the critical texture features for the classification of cell density and media environment conditions in Random forests, which showed the best classification performance in our experimental trials. Among all features, we discovered that the GLCM correlation parameters had a significant measure (i.e., mean decrease in accuracy) in Random forests used to categorize cell density spectra utilizing high–throughput BF images from the ‘DFT Modulus’ of the PPSW (the bior1.1 mother wavelet using the fifth–level decomposition) technique (Supplementary Materials Figure S4a). Note that highly ranked features might be correlated, and therefore not all highly ranked attributes would improve the prediction performance. Therefore, we analyzed the model–agnostic permutation feature importance on the testing set and found that permuting the values of the GLCM correlation feature resulted in the highest decrease in the accuracy score of the model (Supplementary Materials Figure S4b).

**Figure 6:**
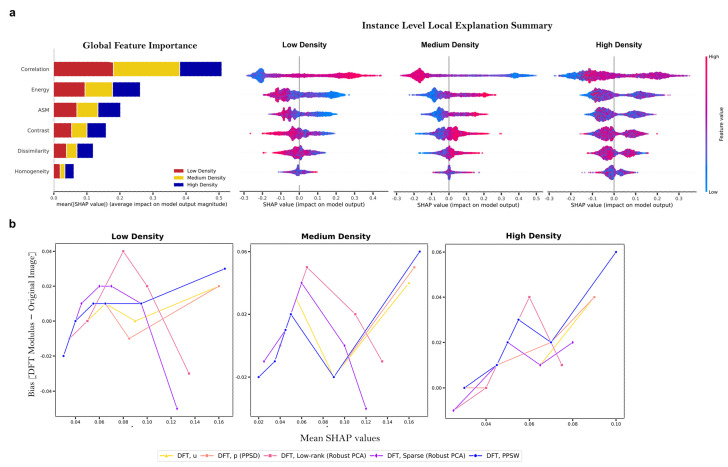
The Shapley Additive exPlanations (SHAP) TreeExplainer–enabled interpretability for key features in the gradient density sample cell density assignments. (**a**, left) Average SHAP values from the ‘DFT Modulus’ of the PPSW technique (the bior1.1 mother wavelet using fifth–level decomposition) applied to BF images. The bar graph shows the correlation between the mean absolute SHAP values and the magnitude of the output generated by Random forests. (**a**, right) Beeswarm plots of texture feature sets for density classes, with dots representing class assignments and colors indicating feature value variations and class correlations. For example, high GLCM correlation values often yield positive SHAP values and low–density spectra classification. (**b**) Mean of differences (=bias) between the ‘DFT Modulus’ transform applied to the decomposition of images and original BF images using average SHAP values from Random forest classifiers.

Aside from the global feature importance, we investigated the classifiers using average SHAP values to identify which features contributed the most to a particular class assignment. Figure 6a presents the SHAP bar plot for the explainability analysis of the cell density spectra classification. It shows the mean of the absolute SHAP values obtained from high–throughput BF images with the ‘DFT Modulus’ of the PPSW (the bior1.1 mother wavelet using fifth–level decomposition) technique (i.e., the average impact of the feature on the model output magnitude). In addition, the SHAP summary plot, also known as the beeswarm summary plot, can provide both global and local important scores, allowing for a more detailed visual summary of each density class assignment in the gradient density samples, as illustrated in Figure 6a. Each dot in the summary plot indicates a sample plotted against its impact on the model output, with samples colored blue indicating a low quantity of texture features and red samples denoting a high abundance. We observed that high feature values of the GLCM correlation parameters commonly had positive SHAP values, promoting the model to classify low–density spectra. In contrast, medium–density spectra had negative SHAP values with higher GLCM correlation features. Furthermore, in order to compare the classification performance of image decomposition and/or restoration techniques for each class cell density assignment, we computed the mean of differences (=bias) between the ‘DFT Modulus’ transform applied to the decomposition of images and original BF images using average SHAP values from Random forest classifiers (Figure 6b). The result showed that between the ‘DFT Modulus’ transform of the PPSW (the bior1.1 mother wavelet using fifth–level decomposition) and of the periodic component, the PPSD technique had a stronger bias for higher average SHAP values, allowing us to make a significant contribution to classification across all samples.

**Figure 7:**
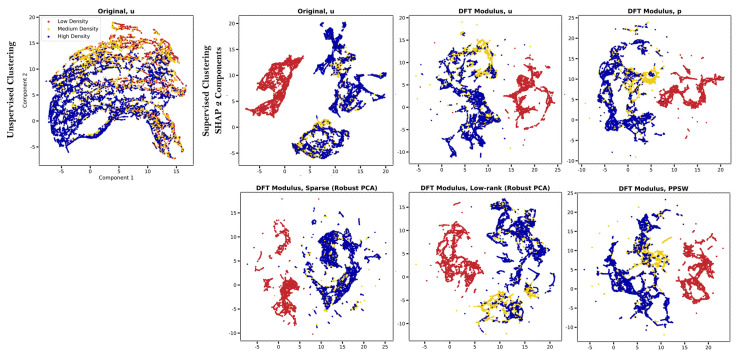
Supervised clustering using SHAP values for the precise, label–free classification of cell density in human MSCs. The raw data (**top left**) and average SHAP values from Random forests (**right** panels) visualized in a two–dimensional space via UMAP.

Traditional clustering techniques are commonly used for microscopy images to identify subgroups within a population that cluster together. Unsupervised learning frequently struggles to establish meaningful structures because such analyses bias samples based on the magnitude of their distributions, regardless of the information contained in the raw data [49]. In our experimental trials, we were unable to distinguish cell density and media environment subgroups from the original BF images, which were projected in a two–dimensional space using UMAP (Figure 7 and Supplementary Materials Figure S5; top left panels). On the other hand, we applied a supervised clustering approach to convert the raw data into SHAP values obtained from Random forests in order to improve the classification performance of the stem cell manufacturing characterization from label–free images. We realized that the SHAP embedding plots of the ‘DFT Modulus’ transform applied to BF images produced highly differentiated clusters, particularly the ‘DFT Modulus’ transform of the PPSW (the bior1.1 mother wavelet using fifth–level decomposition) for cell density spectra and the periodic component, PPSD, for cell media environment conditions (Figure 7 and Supplementary Materials Figure S5; right panels).

## 4. Discussion

Cell Therapies (CTs) are regenerative medicines administered to patients in the form of living cells. Stem cells represent a novel therapeutic approach for the treatment of many chronic diseases, but routine CT manufacturing remains a key barrier to their widespread clinical usage [6,7,8]. In particular, the absence of rapid cell analytics to identify and categorize the stem cell behavior lies at the heart of the manufacturing challenge. To assess the therapeutic quality of stem cells while they are being expanded and before transplantation, it is required to monitor stem cell identity, health, and robustness (ability to deal with change or process intervention). Many modern cell assays rely primarily on chemical staining to identify critical cell components or quantify functional properties. However, staining is costly, time–consuming, and labor–intensive and tends to damage cells. In this study, we have presented an analytical machine learning (ML) workflow for the rapid and efficient classification of high–throughput bright–field (BF) images of stem cells in the context of CT manufacturing. By comparing our proposed workflow with other existing methodology in the field [32,48], we demonstrated its advantages in terms of performance metrics, interpretability, and potential for wider applications.

Our study offers several significant advancements, which are as follows:

- Introduction of the Periodic Plus Smooth Wavelet Transform (PPSW) technique for image decomposition and restoration, minimizing the potential adverse impact of artifact structures on the ML classification accuracy. This technique allows for better feature extraction and representation, leading to higher performance in classification tasks.
- Utilization of a variety of tree–based ML models, specifically Random forest classifiers, for accurate stem cell classification based on label–free cell images. These models offer a fast and efficient way to extract rich biological insights from the images without the need for extensive pre–processing or data augmentation.
- Application of supervised clustering using Shapley Additive exPlanations (SHAP) values, offering both local and global interpretations, to enhance the understanding of CT bioprocesses. We demonstrated that supervised clustering using average SHAP values, obtained from the ‘DFT Modulus’ of the PPSW and of the periodic component, i.e., PPSD techniques applied to original BF images, in the tree–based ML model produced highly distinct clusters of human Mesenchymal Stem Cells (MSCs) corresponding to diverse cell culture conditions. This approach provides a clear understanding of the relationships between different features and their importance in the classification process, making it easier for researchers and practitioners to optimize their workflows.
- Reduction of noise and improved feature properties, allowing models to achieve encouraging accuracy with a limited dataset of approximately 1000 images. This advantage suggests that our proposed workflow can be effective even with relatively small datasets, lowering the barrier to entry for researchers and companies looking to adopt these techniques.
- Demonstration of reasonable computational burden and time for our PPSW technique across different hardware configurations, highlighting the efficiency of our approach. Our PPSW technique showcases reasonable computational burden and time across different hardware configurations, with the GPU server taking 0.115 s for the original image and 0.33 s for the bior2.4 mother wavelet type, while the laptop computer we used took 0.23 s and 0.75 s, respectively, for processing a single image and extracting the six Haralick texture feature sets with noise reduction. This efficiency enables our workflow to be applied in various settings, from high–end research facilities to smaller labs with limited computational resources.

To evaluate the feasibility of our proposed analytical workflow, we used a dataset of images of human MSCs obtained from multiple donors, cultured in various media backgrounds, and under a range of bioprocess conditions. We found that the results of the ‘DFT Modulus’ transform applied to the decomposition of BF images revealed significantly higher descriptive statistics than the original BF images, making it simpler to classify cell density or media background across gradient density samples. Random forest classifiers produced the highest accuracy stem cell classification outcomes. In addition, the ‘DFT Modulus’ transform of the PPSW (the bior1.1 mother wavelet using fifth–level decomposition) for cell density spectra and the periodic component, PPSD for cell media environment conditions, provided superior performance in comparison to all other competitors of concern in this paper.

It is worth noting that the proposed workflow was applied to a relatively small dataset. Challenges related to larger sample sizes may arise, such as increased computational requirements, but our method shows promising results in terms of computational efficiency on various hardware configurations. Further investigation is needed to validate the scalability of our workflow to larger datasets and diverse cell types.

For ML interpretability, the use of SHAP values in our study resulted in improved accuracy, which could be attributed to the advantages provided by the SHAP TreeExplainer method in terms of rescaling the data and enhancing feature relevance. TreeSHAP computed exact SHAP values using its conditional expectation method for each class assignment, which is computationally less expensive than KernelSHAP [50]. Our study not only highlights global feature importance but also showcases the potential of supervised clustering with SHAP values in Random forests. This approach enables the identification of distinct subgroups and enhances explainability by evaluating the significance of individual texture feature sets.

With deeper application–focused examination of the features and interpretation of the ML model within the context of our dataset, we can effectively demonstrate the strengths of our approach and highlight the types of insights it can offer. For example, discussing the best features across various classes and their implications for the classifications present in the data can provide a better understanding of the biological insights that can be derived from our workflow. This discussion could also highlight potential directions for future research, such as investigating the relationships between specific features and stem cell behavior or exploring new feature extraction methods to further improve classification performance. We acknowledge that our study has certain limitations, some of which are specific to the dataset. In addition, our results may be influenced by factors such as data quality, image resolution, and potential bias during the pre–processing of the dataset, which highlights the importance of addressing these issues to provide the robustness and generalizability of our workflow. In the future, we intend to test our proposed analytical workflow on similar large–scale datasets obtained from different specifications of high–throughput microscopy imaging systems to evaluate the generalizability of our approach. This will allow us to refine our methods and address potential limitations, ultimately leading to more robust and effective tools for stem cell research and CT manufacturing.

In conclusion, we aimed to establish an analytical ML workflow with high performance metrics to fully characterize stemness and cell quality from high–throughput BF images in CT manufacturing. Given its local and global interpretations and high fidelity, supervised clustering approach using SHAP values, obtained from the ‘DFT Modulus’ applied to the decomposition of BF images in the trained ML model, would improve the interpretation of CT bioprocesses. Our innovative, label–free approach will enhance stem cell manufacturing through faster and richer process analytics, thereby making stem cell therapies accessible to all. As our approach continues to be refined and tested on a wider range of datasets and applications, we anticipate that it will contribute significantly to the advancement of regenerative medicine, improving patient outcomes, and driving innovation in the development of novel therapies and treatments.

## Figures and Tables

**Table 1:** Comparative analysis of GLCM texture features in original bright–field samples across diverse density and media environment conditions (Descriptive statistics: (mean (standard deviation) or median (interquartile range; 25–75%)), Statistical results: (*p* value)).

Media	Variables	Gradient Density Spectra			*p* Value_+_
		Low	Medium	Hight	
LSC	Homogeneity	0.741 (0.022) ^α^	0.715 (0.695–0.735)	0.695 (0.674–0.720)	<0.001
	Contrast	0.727 (0.617–0.851) ^β^	0.943 (0.755–1.152)	1.119 (0.848–1.322)	
	Correlation	0.616 (0.456–0.766)	0.580 (0.408–0.738)	0.571 (0.387–0.715)	
	Dissimilarity	0.549 (0.512–0.592)	0.626 (0.563–0.687)	0.685 (0.604–0.748)	
	Energy	0.345 (0.308–0.382)	0.326 (0.296–0.362)	0.312 (0.278–0.341)	
	ASM	0.118 (0.095–0.146)	0.106 (0.087–0.131)	0.098 (0.077–0.116)	
SC	Homogeneity	0.748 (0.738–0.759)	0.718 (0.698–0.736)	0.675 (0.648–0.701)	<0.001
	Contrast	0.711 (0.615–0.824)	1.007 (0.781–1.248)	1.339 (0.981–1.927)	
	Correlation	0.622 (0.461–0.803)	0.568 (0.385–0.734)	0.557 (0.342–0.703)	
	Dissimilarity	0.532 (0.504–0.565)	0.628 (0.565–0.691)	0.748 (0.650–0.836)	
	Energy	0.339 (0.306–0.393)	0.330 (0.287–0.369)	0.294 (0.258–0.328)	
	ASM	0.115 (0.093–0.155)	0.109 (0.082–0.136)	0.087 (0.067–0.107)	
SF	Homogeneity	0.744 (0.731–0.755)	0.709 (0.684–0.733)	0.671 (0.644–0.701)	<0.001
	Contrast	0.718 (0.616–0.859)	0.942 (0.736–1.126)	1.212 (0.882–1.469)	
	Correlation	0.652 (0.505–0.806)	0.567 (0.392–0.734)	0.582 (0.376–0.721)	
	Dissimilarity	0.542 (0.509–0.582)	0.636 (0.567–0.705)	0.742 (0.633–0.826)	
	Energy	0.337 (0.295–0.376)	0.322 (0.278–0.354)	0.293 (0.257–0.326)	
	ASM	0.114 (0.087–0.141)	0.104 (0.078–0.126)	0.086 (0.066–0.106)	

^α^: mean (standard deviation), ^β^: median (interquartile range). _+_: *p* value is considered to be significant if *p* < 0.05.

**Table 2:** Performance metrics for the top three tree–based machine learning models in cell density spectra categorization, using high–throughput bright–field images (as our baseline scenario) as well as images with image decomposition and/or restoration techniques in the Gradient Density dataset.

Variables	Model	Percentage Split			
		Accuracy	Precision	Recall	F1–Score
Original, *u* Baseline	LightGBM	73.945	73.155	73.770	73.928
	XGBoost	74.796	74.177	74.635	74.314
	Random Forest	80.129	79.797	80.100	79.887
DFT modulus, *u*	XGBoost	87.268	87.404	87.202	87.295
	Decision Tree	88.727	88.694	88.702	88.696
	Random Forest	92.826	92.875	92.792	92.832
DFT modulus, *u_low__–__rank_*	XGBoost	87.216	87.363	87.168	87.247
	Decision Tree	90.116	90.099	90.172	90.125
	Random Forest	92.322	92.308	92.335	92.321
DFT modulus, *u_sparse_*	XGBoost	86.625	86.784	86.360	86.526
	Decision Tree	88.848	88.786	88.763	88.770
	Random Forest	93.556	93.569	93.452	93.505
DFT modulus, *p*	LightGBM	88.154	88.173	88.078	88.117
	Decision Tree	91.489	91.443	91.426	91.432
	Random Forest	94.511	94.499	94.442	94.476
DFT modulus, *PPSW*	XGBoost	88.917	89.042	88.839	88.932
	Decision Tree	90.846	90.775	90.821	90.797
	Random Forest	94.754	94.744	94.729	94.734

*u*: original image, *u_low_*_–_*_rank_*: low–rank matrix decomposition via Robust PCA. *u_sparse_*: sparse decomposition via Robust PCA. *p*: periodic component of the image with periodic plus smooth image decomposition (PPSD). *PPSW:* Periodic plus smooth wavelet transform technique.

**Table 3:** Performance metrics for the top three tree–based machine learning models in cell media environment spectra categorization within the Gradient Density dataset.

Variables	Model	Percentage Split			
		Accuracy	Precision	Recall	F1–Score
Original, *u* Baseline	Decision Tree	71.947	71.777	71.782	71.767
	XGBoost	72.295	72.204	72.210	72.217
	Random Forest	80.146	80.041	80.085	80.058
DFT modulus, *u*	XGBoost	80.684	80.727	80.625	80.649
	Decision Tree	85.861	85.863	85.874	85.868
	Random Forest	90.290	90.318	90.294	90.306
DFT modulus, *u_low__–__rank_*	XGBoost	78.426	78.555	78.320	78.402
	Decision Tree	83.933	83.926	83.858	83.889
	Random Forest	89.196	89.219	89.153	89.182
DFT modulus, *u_sparse_*	XGBoost	85.948	86.306	85.532	85.721
	Decision Tree	89.387	89.344	89.355	89.312
	Random Forest	93.677	93.705	93.564	93.625
DFT modulus, *p*	Decision Tree	90.099	90.386	90.377	90.381
	XGBoost	90.603	90.886	90.976	90.898
	Random Forest	93.260	93.440	93.518	93.466
DFT modulus, *PPSW*	XGBoost	81.414	81.468	81.276	81.351
	Decision Tree	86.434	86.394	86.468	86.425
	Random Forest	91.437	91.403	91.436	91.419

*u*: original image, *u_low_*_–_*_rank_*: low–rank matrix decomposition via Robust PCA. *u_sparse_*: sparse decomposition via Robust PCA. *p*: periodic component of the image with periodic plus smooth image decomposition (PPSD). *PPSW:* Periodic plus smooth wavelet transform technique.

## Data Availability

All information collected from this study has been kept confidential. The data presented in this study are available upon request to the corresponding author. Additionally, the authors are pleased to provide the source–code for this study upon request.

## Supplementary Materials

The following supporting information can be downloaded at: https://www.mdpi.com/article/10.3390/cells12101384/s1, Figure S1: Schematic representation of the machine learning pipeline employed in this study; Figure S2: Human Mesenchymal Stem Cells (MSCs) from bone marrow in different media environments—Serum Free (SF, left), Low–Serum Containing (LSC, middle), and Serum Containing (SC, right). Upper panels display overlay images with Hoechst stain (red) and Cell mask (green). Lower panels present corresponding bright–field images for each media environment, showcasing the distinct morphological characteristics of human MSCs. Abbreviations: FBS—Fetal Bovine Serum; FGF—Fibroblast Growth Factor 2; Figure S3: Illustrative examples of discrete wavelet families employed in this study; Figure S4: Random Forest global feature importance (left) and permutation importance (right) scores derived from the ‘DFT Modulus’ of the Periodic Plus Smooth Wavelet (PPSW) transform (with the bior1.1 mother wavelet using fifth–level decomposition) applied to bright–field images; Figure S5: Supervised clustering using SHAP values facilitates precise, label–free classification of cell media environment in human MSCs. The raw data (top left) and average SHAP values from Random Forests (right panels) projected in two–dimensional space via UMAP; Table S1: Performance metrics of Random Forest classifiers for cell density spectra using discrete second–level mother wavelet functions; Table S2: Performance metrics of Random Forest classifiers for cell media environment spectra using discrete second–level mother wavelet functions; Table S3: Performance metrics of various state-of-the-art tree–based machine learning models for classifying cell density spectra, utilizing high–throughput bright–field images (our baseline scenario) as well as images with image decomposition and/or restoration techniques in the Gradient Density dataset; Table S4: Performance metrics of various state-of-the-art tree–based machine learning models for classifying cell media environment spectra within the Gradient Density dataset.
