# Supplementary material for: PPSW–SHAP: Towards Interpretable Cell Classification Using Tree-Based SHAP Image Decomposition and Restoration for High-Throughput Bright-Field Imaging
Source: Cells. 2023 May 13;12(10):1384. doi: 10.3390/cells12101384 (PMC10216550; doi:10.3390/cells12101384)
Supplement: Supplementary file 1 [file cells-12-01384-s001.zip › SupplementaryFigures-cells-2277043.pdf]

Supplementary Figures

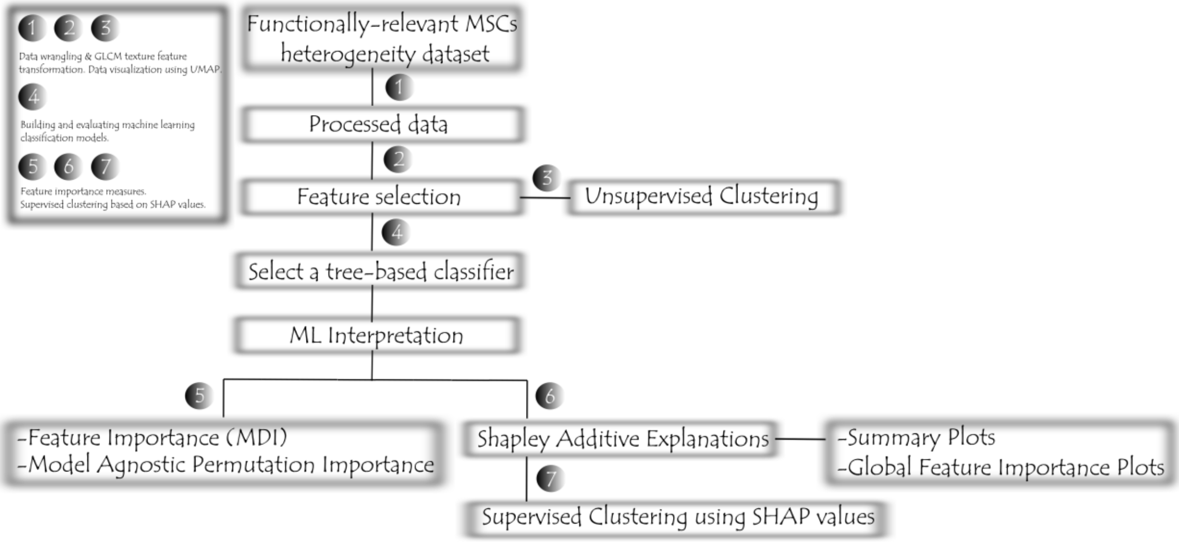

Figure S1. Schematic representation of the machine learning pipeline employed in this study.

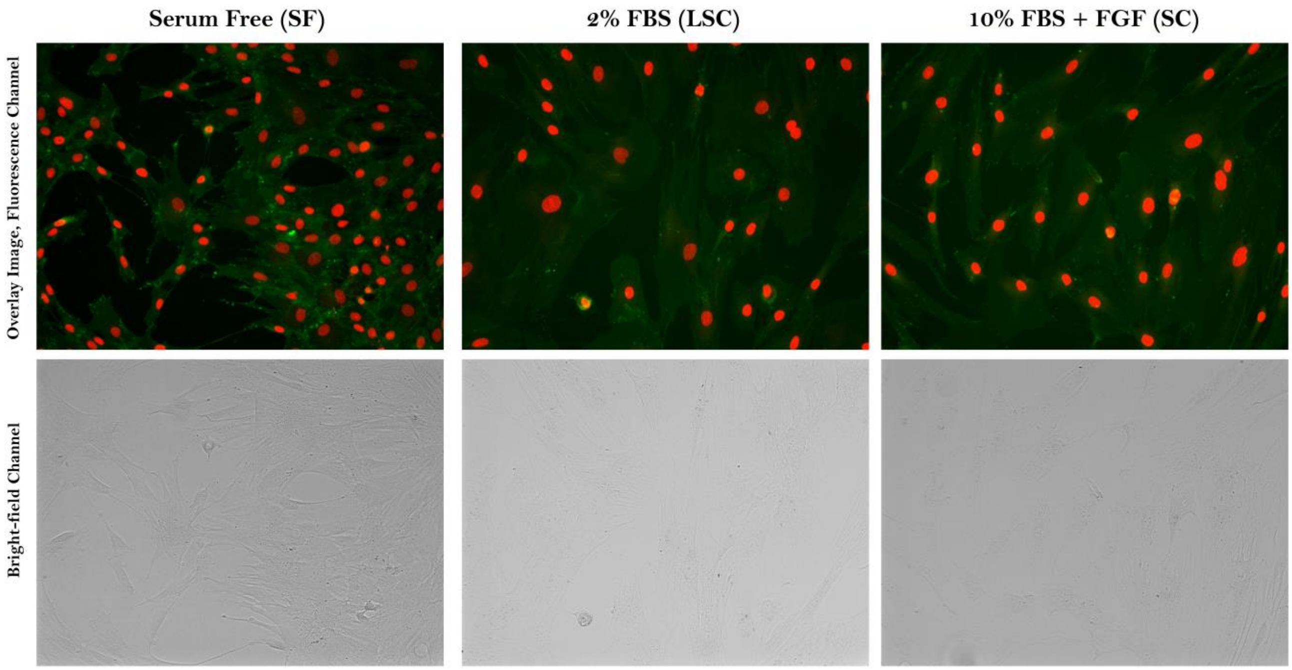

Figure S2. Human Mesenchymal Stem Cells (MSCs) from bone marrow in different media environments − Serum Free (SF, left), Low−Serum Containing (LSC, middle), and Serum Containing (SC, right). Upper panels display overlay images with Hoechst stain (red) and Cell mask (green). Lower panels present corresponding bright−field images for each media

environment, showcasing the distinct morphological characteristics of human MSCs. **Abbreviations**: FBS − Fetal Bovine Serum; FGF − Fibroblast Growth Factor 2.

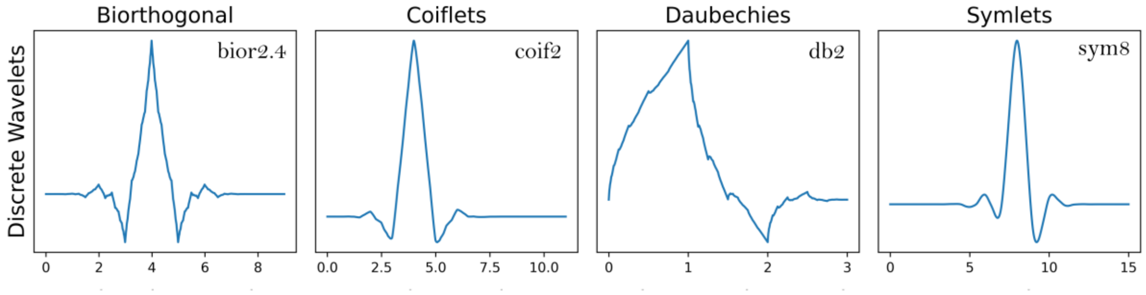

Figure S3. Illustrative examples of discrete wavelet families employed in this study.

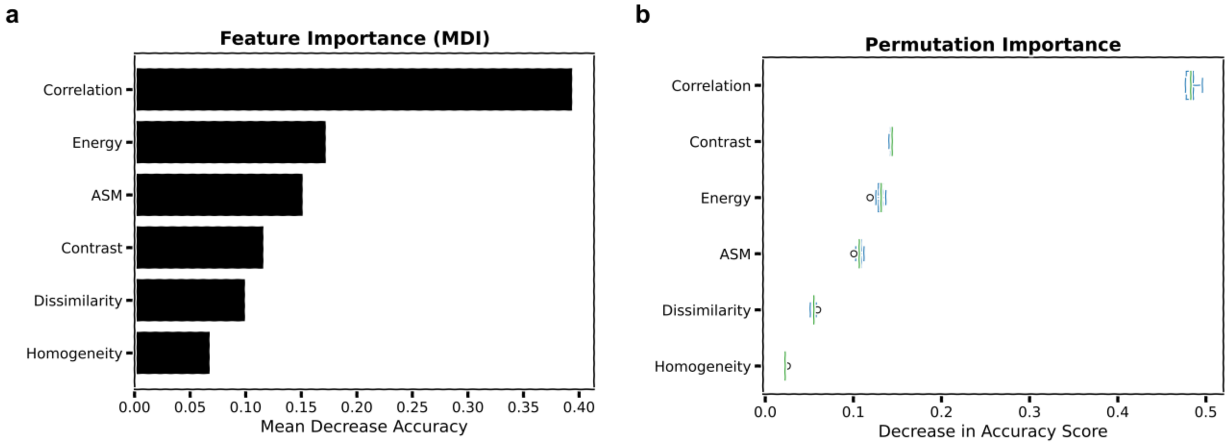

Figure S4. Random Forest global feature importance (left) and permutation importance (right) scores derived from the 'DFT Modulus' of the Periodic Plus Smooth Wavelet (PPSW) transform (with the bior1.1 mother wavelet using fifth−level decomposition) applied to bright−field images.

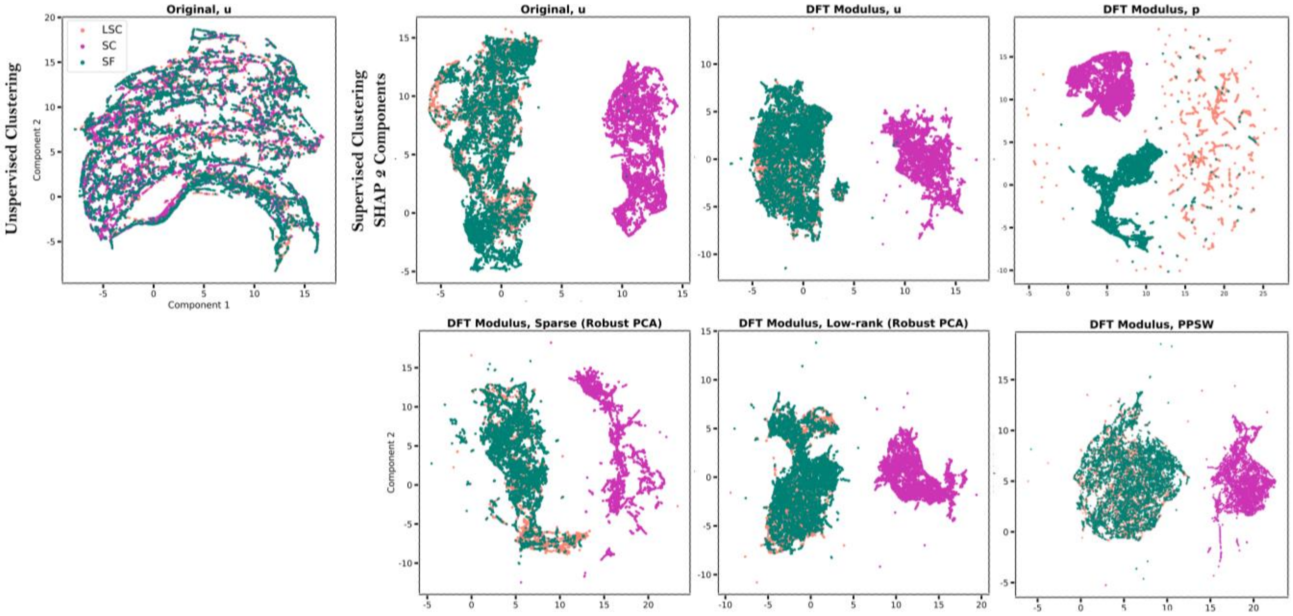

Figure S5. Supervised clustering using SHAP values facilitates precise, label−free classification of cell media environment in human MSCs. The raw data (top left) and average SHAP values from Random Forests (right panels) projected in two−dimensional space via UMAP.
