# Supplementary material for: PPSW–SHAP: Towards Interpretable Cell Classification Using Tree-Based SHAP Image Decomposition and Restoration for High-Throughput Bright-Field Imaging
Source: Cells. 2023 May 13;12(10):1384. doi: 10.3390/cells12101384 (PMC10216550; doi:10.3390/cells12101384)
Supplement: Supplementary file 1 [file cells-12-01384-s001.zip › SupplementaryTables-cells-2277043.pdf]

Supplementary Tables

Table S1. Performance metrics of Random Forest classifiers for cell density spectra using discrete second−level mother wavelet functions.

		Percentage Split			
Method	*Variables*	*Accuracy*	*Precision*	*Recall*	*F1-score*
DFT modulus, PPSW	*bior*1.1	91.836	91.774	91.766	91.760
	*bior*2.4	91.523	91.480	91.452	91.456
	*bior*6.8	91.089	90.935	90.944	90.935
	*coif*1	91.089	91.013	90.975	90.992
	*coif*2	91.245	91.154	91.157	91.153
	*coif*5	88.813	88.654	88.634	88.616
	*db*2	91.141	91.195	91.025	91.103
	*db*10	89.561	89.437	89.445	89.439
	*sym*2	91.141	91.195	91.025	91.103
	*sym*8	89.873	89.723	89.737	89.720

Table S2. Performance metrics of Random Forest classifiers for cell media environment spectra using discrete second−level mother wavelet functions.

		Percentage Split			
Method	*Variables*	*Accuracy*	*Precision*	*Recall*	*F1-score*
DFT modulus, PPSW	*bior*1.1	88.171	88.193	88.137	88.162
	*bior*2.4	87.528	87.574	87.456	87.506
	*bior*6.8	86.121	86.091	86.175	86.128
	*coif*1	87.146	87.203	87.105	87.144
	*coif*2	87.372	87.373	87.296	87.325
	*coif*5	85.305	85.292	85.288	85.290
	*db*2	87.827	87.997	87.878	87.929
	*db*10	85.409	85.403	85.353	85.376
	*sym*2	87.928	87.997	87.878	87.929
	*sym*8	86.694	86.664	86.667	86.663

Table S3. Performance metrics of various state-of-the-art tree–based machine learning models for classifying cell density spectra, utilizing high−throughput bright−field images (our baseline scenario) as well as images with image decomposition and/or restoration techniques in the Gradient Density dataset.

		Percentage Split			
Variables	*Model*	*Accuracy*	*Precision*	*Recall*	*F1-score*
Original, u **Baseline**	AdaBoost	68.074	67.270	67.823	67.490
	GBDT	68.838	67.704	68.499	67.926
	LightGBM	73.945	73.155	73.770	73.928
	HistGBDT	74.622	74.016	74.511	74.171
	Decision Tree	74.587	74.531	74.603	74.561
	XGBoost	74.796	74.177	74.635	74.314
	Random Forest	80.129	79.797	80.100	79.887
DFT modulus, u	AdaBoost	68.404	68.759	68.307	68.512
	GBDT	71.131	71.999	71.028	71.430
	LightGBM	84.263	84.592	84.238	84.387
	HistGBDT	85.756	85.901	85.775	85.831
	Decision Tree	88.727	88.694	88.702	88.696
	XGBoost	87.268	87.404	87.202	87.295
	Random Forest	92.826	92.875	92.792	92.832
DFT modulus, $u_{low-rank}$	AdaBoost	69.706	69.000	69.706	69.275
	GBDT	73.476	74.031	73.491	73.719
	LightGBM	85.756	85.945	85.706	85.804
	HistGBDT	86.729	86.886	86.714	86.788
	Decision Tree	90.116	90.099	90.172	90.125
	XGBoost	87.216	87.363	87.168	87.247
	Random Forest	92.322	92.308	92.335	92.321
DFT modulus, u_{sparse}	AdaBoost	58.294	58.898	57.719	58.047
	GBDT	60.570	61.543	59.559	59.778
	LightGBM	82.439	82.590	82.054	82.256
	HistGBDT	85.739	85.821	85.417	85.580
	Decision Tree	88.848	88.786	88.763	88.770
	XGBoost	86.625	86.784	86.360	86.526
	Random Forest	93.556	93.569	93.452	93.505
DFT modulus, p	AdaBoost	69.394	69.133	69.069	68.949
	GBDT	71.391	71.743	71.143	71.370
	LightGBM	88.154	88.173	88.078	88.117
	HistGBDT	82.039	82.264	82.017	82.123
	Decision Tree	91.489	91.443	91.426	91.432
	XGBoost	86.729	86.727	86.630	86.648
	Random Forest	94.511	94.499	94.442	94.476
DFT modulus, *PPSW*	AdaBoost	68.629	68.226	68.414	68.281
	GBDT	71.408	71.578	71.174	71.331
	LightGBM	86.902	87.052	86.812	86.918
	HistGBDT	88.571	88.605	88.516	88.557
	Decision Tree	90.846	90.775	90.821	90.797
	XGBoost	88.917	89.042	88.839	88.932
	Random Forest	94.754	94.744	94.729	94.734

Table S4. Performance metrics of various state-of-the-art tree–based machine learning models for classifying cell media environment spectra within the Gradient Density dataset.

		Percentage Split			
Variables	*Model*	*Accuracy*	*Precision*	*Recall*	*F1-score*
Original, u	AdaBoost	55.324	56.199	55.488	55.390
Baseline	GBDT	57.634	57.732	57.564	57.570
	LightGBM	69.654	69.700	69.712	69.609
	HistGBDT	70.019	70.034	70.083	69.971
	Decision Tree	71.947	71.777	71.782	71.767
	XGBoost	72.295	72.204	72.210	72.217
	Random Forest	80.146	80.041	80.085	80.058
DFT modulus, u	AdaBoost	50.860	50.706	50.986	50.749
	GBDT	53.135	53.155	52.986	52.882
	LightGBM	75.682	75.084	75.489	75.611
	HistGBDT	78.079	78.121	78.030	78.053
	Decision Tree	85.861	85.863	85.874	85.868
	XGBoost	80.684	80.727	80.625	80.649
	Random Forest	90.290	90.318	90.294	90.306
DFT modulus, $u_{low-rank}$	AdaBoost	48.602	48.223	48.642	47.897
	GBDT	51.711	51.521	51.651	50.935
	LightGBM	75.022	75.817	74.865	74.920
	HistGBDT	77.870	78.020	77.808	77.874
	Decision Tree	83.933	83.926	83.858	83.889
	XGBoost	78.426	78.555	78.320	78.402
	Random Forest	89.196	89.219	89.153	89.182
DFT modulus, u_{sparse}	AdaBoost	59.823	58.626	58.444	56.658
	GBDT	61.560	60.763	60.263	59.363
	LightGBM	80.928	81.618	80.255	80.382
	HistGBDT	83.307	83.603	82.834	82.962
	Decision Tree	89.387	89.344	89.355	89.312
	XGBoost	85.948	86.306	85.532	85.721
	Random Forest	93.677	93.705	93.564	93.625
DFT modulus, p	AdaBoost	65.086	62.103	64.073	54.660
	GBDT	84.523	85.157	85.199	85.021
	LightGBM	89.682	90.008	90.092	90.011
	HistGBDT	89.977	90.290	90.375	90.295
	Decision Tree	90.099	90.386	90.377	90.381
	XGBoost	90.603	90.886	90.976	90.898
	Random Forest	93.260	93.440	93.518	93.466
DFT modulus, $PPSW$	AdaBoost	50.009	49.914	49.998	49.927
	GBDT	53.483	53.458	53.409	53.354
	LightGBM	77.506	77.666	77.307	77.398
	HistGBDT	80.372	80.498	80.241	80.329
	Decision Tree	86.434	86.394	86.468	86.425
	XGBoost	81.414	81.468	81.276	81.351
	Random Forest	91.437	91.403	91.436	91.419
